# Trends in Management of Radial Head and Olecranon Fractures

**DOI:** 10.2174/1874325001711010239

**Published:** 2017-03-31

**Authors:** Matthew Motisi, Jennifer Kurowicki, Derek D. Berglund, Jacob J. Triplet, Shanell Disla, Timothy Niedzielak, Jonathan C. Levy

**Affiliations:** 1 Broward Health Medical Center, 1800 NW 49th Street Fort Lauderdale, FL 33309, USA; 2 Holy Cross Orthopedic Institute, 5597 N. Dixie Highway Fort Lauderdale, FL 33334, USA; 3 Doctors Hospital- Ohio Health, 5100 West Broad Street, Columbus, OH 43228, USA; 4 Nova Southeastern University, College of Osteopathic Medicine, 3301 College Avenue Fort Lauderdale, FL 33314, USA

**Keywords:** Elbow fracture management, Non-operative management, Olecranon fracture, Open reduction internal fixation, Radial head arthroplasty, Radial head fracture, Radial neck fracture

## Abstract

**Background:**

Advancement in surgical techniques and implants has improved the ability to manage radial head and olecranon fractures. However, trends in management of these fractures are largely unstudied.

**Objective:**

This purpose of this study is to evaluate management trends for these common fractures.

**Methods:**

A retrospective review of a comprehensive Humana database was performed using Pearl Diver supercomputer (Warsaw, IN, USA) for radial head and neck (RHNF) and olecranon fractures (OF) between 2007 and 2014. Treatment methods including open reduction internal fixation (ORIF), radial head arthroplasty (RHA), and non-operative treatment were reviewed. Total reported incidence of office visits and utilization of each treatment modality were investigated. Sub-analysis with stratification by age 15-74 and greater than 75-years was performed for OF.

**Results:**

A total of 10,609 OF and 20,400 RHNF were identified between 2007 and 2014. A significant trend increase in the annual incidence of RHNF (266 cases/year, p<0.001) and OF (133.9 cases/year, p=0.001) was observed. A significant trend *increase* in annual percent utilization of RHA (0.22% per year, p=0.011) and a significant trend *decrease* in the annual percent utilization of ORIF (-1.0% per year, p=0.004) and non-operative management (-0.53% per year, p=0.046) was observed for RHNF. A significant trend *increase* was observed in percent utilization (0.40% per year, p=0.022) for OF non-operative management, especially in patients over 75 years (66% per year, p=0.034).

**Conclusion:**

The percentage of patients being treated with RHA is increasing. Non-operative management of OF has increased, specifically in the patients who are over 75 years.

## INTRODUCTION

Fractures of the proximal ulna and radius are the most commonly observed elbow fractures [1-3]. While minimally-displaced, stable fractures are typically managed with non-operative treatment, those that are significantly displaced or comminuted are commonly treated with surgical intervention [4, 5].

Displaced fractures of the olecranon have historically been managed with variable methods of osteosynthesis [6-11]. However, there has been a more recent emphasis on non-operative treatment of displaced olecranon fractures (OF) in the elderly [12-14]. Displaced radial head fractures have historically been managed with osteosynthesis [15, 16] or radial head excision [17]. More recently, osteosynthesis has been suggested when the displaced fragments result in a loss of forearm rotation or are combined with ligamentous injuries [18]. Furthermore, those fractures that contain three or more fragments are often managed with radial head arthroplasty (RHA), as osteosysnthesis has been shown to result in high rates of unsatisfactory outcomes [19].

While the literature has helped to outline those fractures that might best be treated with surgery, and innovations in surgical technique and surgical implants have continued to improve, national trends have not been examined to determine the impact of these advances. The purpose of this study is to examine the national trends for management of two common elbow fractures-1) radial head and neck fractures (RHNF); 2) olecranon fractures – using the Humana claims database between 2007 and 2014. We hypothesize a gradual increase in patients being treated with RHA for RHNF and non-operative management for OF compared with ORIF for these fractures during this time period.

## METHODS

A retrospective review of the entire Humana database for radial head and neck fractures as well as olecranon fractures in patients over 15 years old was conducted through the use of the Pearl Diver supercomputer (Warsaw, IN, USA). This software is a publicly available Health Insurance Portability and Accountability Act-compliant database that compiles the entire Humana files into a server. Data compiled within the Humana database are derived from a combination of Medicare/Medicare Advantage and private-payers yielding 18,620,198 patients from 2007-2014. Pearl Diver software provides access to the Humana registries with the use of Current Procedural Terminology (CPT; American Medical Association, Chicago, IL, USA) codes, as well as, International Classification of Disease Ninth Edition (ICD-9) codes.

A query of the Humana database was performed for OF using ICD-9 codes 813.01 (closed fracture of olecranon process), 813.11 (open fracture of olecranon process), 813.04 (other and unspecified closed fracture of proximal end of ulna, alone), and 813.14 (other and unspecified open fractures of the proximal end of ulna, alone). Open reduction with internal fixation and non-operative treatment of OF was identified *via* ICD-9 and CPT codes (Table **1**). A sub-analysis stratification of OF by age was untaken; patients between the ages of 15-74 years (Group A) and greater than 75 years (Group B) were reviewed.

RHNF were queried *via* the Humana database using ICD-9 codes 813.05 (closed fracture of head of radius), 813.15 (open fracture of neck of radius), 813.06 (closed fracture of neck of radius), 813.16 (open fracture of neck of radius), 813.07 (other and unspecified closed fracture of proximal end of radius, alone) and 813.17 (other and unspecified open fracture of proximal end of radius, alone). Radial head arthroplasty (RHA), ORIF, and non-operative treatment of RHNF were identified using ICD-9 and CPT codes (Table **1**).

The incidence of reported OF and RHNF was examined and trend changes for each fracture type were reported. Utilization of non-operative management as well as surgical procedures (ORIF and radial head arthroplasty) was examined to define trend changes. The percent utilization of each treatment modality was also examined to detect any change trends in treatment selections for each fracture type.

Statistical analysis was mainly descriptive to define the incidence of each procedure throughout the study period. Linear regression models were analyzed to examine any trends in incidence of fracture type, incidence of treatment modalities chosen, as well as percent utilization for these treatment modalities using a 95% confidence interval. A p-value of <0.05 was selected to be significant. Moreover, correlational analysis using the Pearson method was used to observe any relationships between trends in incidence and concomitant trends in treatment modalities. These same correlational analyses were carried out for stratified age groups. Statistical analysis was performed with Prism 6 software (GraphPad Software, La Jolla, CA, USA).

## RESULTS

**Fig. (1):**
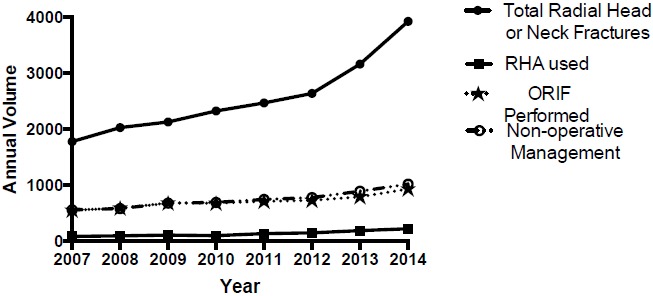
Radial head & neck fractures and management.

**Fig. (2):**
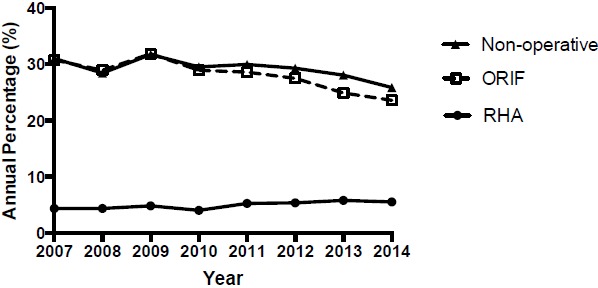
Management of radial head and neck fracture.

A total of 20,400 RHNF were identified in the Humana population between 2007 and 2014 (Table **2**). A significant trend increase in annual reported incidence of RHNF was observed (Fig. **1**); R^2^= 0.88; p<0.001; 95% CI [169.2, 363.2]) at a rate of 266 cases per year. A total of 1,028 RHNF were managed with a RHA with a significant trend increase in the annual percentage of RHNF managed with RHA at a rate of 0.22% per year (Fig. **2**); R^2^= 0.68; p=0.011; 95% CI [0.07, 0.36]). A total of 5,612 RHNF were treated with ORIF with a significant decrease trend in the annual percentage of RHNF fractures managed with ORIF at a rate of -1.0% per year (R^2^= 0.78; p=0.004; 95% CI [-1.53, -0.46]). A total of 5,887 RHNF were managed non-operatively with a significant decrease trend observed in annual percentage of RHNF managed without surgery at a rate of -0.53% per year (R^2^= 0.51; p=0.046; 95% CI [-1.06, -0.01]).

**Fig. (3):**
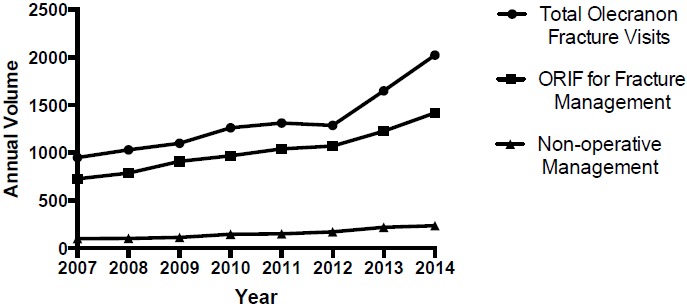
Annual olecranon fractures & management.

**Fig. (4):**
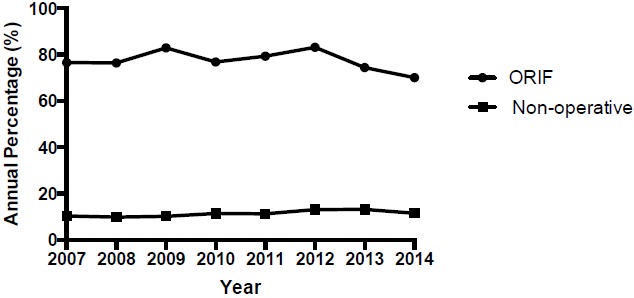
Management of olecranon fractures.

A total of 10,609 OF were identified in the Humana population between 2007 and 2014 (Table **3**). A significant trend increase in the annual incidence of olecranon fractures (Fig. **3**); R^2^= 0.86; p=0.001; 95% CI [78.88, 188.9]) was observed at a rate of 133.9 fractures per year. A total of 8,146 OF were managed with ORIF with no significant trend change in the annual percentage of OF treated with ORIF (R^2^= 0.12; p=0.392; 95% CI [-2.28, 1.03]). A total of 1,225 OF were managed non-operatively with significant trend increase observed in the annual percentage of OF treated without surgery at a rate of 0.40% per year (Fig. **4**); R^2^= 0.61; p=0.022; 95% CI [0.08, 0.72]).

Stratification of olecranon fracture management by age demonstrated differences. Group A (15-74 years) showed a significant trend increase in annual incidence of OF at a rate of 70.92 fractures per year (R^2^= 0.78; p=0.004; 95% CI [32.99, 108.8]), utilization of ORIF at a rate of 50.56 cases per year (R^2^= 0.87; p<0.001; 95% CI [31.06, 70.06]), and utilization of non-operative management, at a rate of 10.29 cases per year (R^2^= 0.85; p=0.001; 95% CI [5.92, 14.65]), of olecranon fractures. These increases in management had a significantly strong correlation to the increase in total olecranon fractures for both ORIF (*r*=0.959; p<0.001) and non-operative management (*r*=0.954; p<0.001). There were no significant trends observed in percentage of olecranon fractures treated with ORIF (R2= 0.01; p=0.835; 95% CI [-2.26, 1.90]) and the percentage managed non-operatively (R^2^= 0.34; p=0.126; 95% CI [-0.11, 0.67]). In Group B (≥75 years), a significant trend increase in annual incidence of OF at a rate of 69.29 fractures per year (R^2^= 0.93; p<0.001; 95% CI [50.86, 87.71]), utilization of ORIF at a rate of 49.42 cases per year (R^2^= 0.95; p<0.001; 95% CI [38.54, 60.29]), and utilization of non-operative management at a rate of 11.35 cases per year (R^2^=0.93; p<0.001; 95% CI [8.27, 14.42]) of OF was seen. Significantly strong correlations were observed between annual incidence of OF and utilization of ORIF (*r=*0.985; p<0.001) and utilization of non-operative treatment (*r*=0.945; p<0.001). No significant trend in percentage of OF managed with ORIF (R^2^= 0.27; p=0.190; 95% CI [-3.30, 0.81]) was seen. However, there was an increase in the annual percentage of OF treated non-operatively at a rate of 0.66% per year (R^2^= 0.56; p=0.034; 95% CI [0.07, 1.25]).

## DISCUSSION

Results of this study show a recent shift in the management of both olecranon and radial head and neck fractures among the Humana population. A significantly increased percentage of patients are being treated with non-operative management for olecranon fractures and radial head arthroplasty is now used more frequently for radial head and neck fractures. Furthermore, in the over-75-year-old patient population, non-operative management of olecranon fractures is increasing.

The current study shows that the incidence of radial head fractures is increasing. Treatment options for these fractures have included non-operative therapy, radial head resection, ORIF, and RHA. Non-operative treatment has historically been recommended for stable, nondisplaced fractures [20, 21]. Radial head resection has been utilized since at least the early 1900s [22] and, with the advent of the Mason classification, was initially recommended for displaced or comminuted fractures [21]. ORIF was later recommended as treatment for comminuted radial head fractures as it was found to have satisfactory outcomes and avoided many of the complications of radial head resection [15, 16]. Management with radial head prostheses were initially recommended for radial head fractures previously treated with radial head excision that resulted in a grossly unstable elbow [23].

Recent findings have re-shaped the management of radial head fractures Table **4**. Radial head resection has mostly fallen out of favor due to the recognition of the radial head as a secondary stabilizer of the elbow and poor outcomes and instability often associated with the procedure [24]. Mason type II fractures have been successfully treated without surgery [20, 25] and ORIF of fractures with 3 or more fragments have been shown to have worse outcomes [19]. Several randomized control trials have shown the superiority of RHA compared to ORIF in terms of post-operative complications and outcome scores for Mason type III and IV fractures [26, 27]. These findings have likely contributed to the trend increase in utilization of RHA (p<0.001) observed in the current study, and likely contributed to the decreasing trends in utilization of ORIF (p=0.004) and non-operative management (p=0.046) for radial head and neck fractures. Additional factors that may have contributed to these trends include the shorter operative time with RHA, improvement of radial head implant technology and surgical techniques, and the increased technical challenge posed by ORIF in comminuted fractures.

Trends in the management of olecranon fractures were found to have changes as well. While no significant change was noted in the percent utilization of ORIF, there were trend increases observed in the percent utilization of non-operative management. This was specifically noted for patients over the age of 75, where the percentage of non-operative treatment was found to have increased. Treatment options for olecranon fractures have historically consisted of non-operative therapy and various methods of fracture fixation including k-wires, tension-band, intra-medullary screw, intra-medullary nail, and plate fixation [7-11]. Complications were found to be linked to fixation methods and thus fragment excision with repair of the triceps insertion was considered as an alternative treatment [6, 28]. Improvements in limiting these complications led to resurgence in the use of ORIF [29]. However, relatively recent articles suggest non-operative treatment as an option for low-demand elderly patients [12-14], thus re-shaping the management of olecranon fractures in this age group (Table **5**). Veras Del Monte *et al.* reviewed non-operative management in 12 elderly patients and reported good short-term clinical outcomes with 92% excellent patient satisfaction and 67% of patients reporting no pain. No patients reported limitations in their daily activities [14]. In a retrospective review of 43 patients with a mean age of 76 years, Duckworth *et al.* reported 72% of patients had excellent or good short-term outcomes, with 23 patients available for long-term follow up and an overall patient satisfaction rating of 91% [12]. These findings have likely contributed to the significant trend increase in the percent utilization of non-operative treatment of olecranon fractures in patients aged over 75 years observed in the current study.

This study is not without limitations. First, the analysis is limited to the Humana database. Selection bias may exist as the Humana patient population may not be representative of the entire country. Additionally, the accuracy of the data is contingent on proper coding practices. More than one indication for common elbow fracture might have been coded (*i.e.*, olecranon fracture and radial head fracture) and there may be overlap in treatment modality (ICD-9 79.32 is ORIF of radius and ulna). In addition, the use of ICD-9 codes allows for less specificity of fracture type or treatment. The major strength of this study relates to the completeness of the data. The PearlDiver database represents complete data for the entire Humana private-payer population.

## CONCLUSION

A shift in management trends for radial head and neck fractures and olecranon fractures among the Humana population has been found in recent years. The percentage of patients being treated with RHA for radial head and neck fractures is increasing. Additionally, non-operative management of olecranon fractures has increased in recent years; more so amongst patients aged 75 years or older.

## Figures and Tables

**Table 1:** Codes used for the query of Humana database.

**Procedure**	**Code**
**ORIF of Olecranon Fracture**	
Open treatment of olecranon process with or without internal or external fixation	CPT 24685
**ORIF of Radial Head and Neck Fractures**	
Open treatment of radial head or neck fracture with or without internal fixation or radial head excision	CPT 24665
ORIF of Olecranon Fracture OR Radial Head and Neck Fracture*	
Internal fixation of bone without fracture reduction, radius and ulna	ICD-9: 78.53
Open reduction of fracture with internal fixation, radius and ulna	ICD-9: 79.32
Open reduction of separated epiphysis, radius and ulna	ICD-9: 79.52
**Non- operative Management of Olecranon Fracture**	
Closed treatment of olecranon process; without manipulation	CPT 24670
Closed treatment of olecranon process; with manipulation	CPT 24675
**Radial Head Arthroplasty**	
Arthroplasty radial head; with implant	CPT 24366
Open treatment of radial head or neck fracture with or without internal fixation or radial head excision; with radial head prosthetic replacement	CPT 24666
Other repair of elbow	ICD-9: 81.85
**Non- operative Management of Radial Head and Neck Fractures**	
Closed treatment of radial head or neck fracture; without manipulation	CPT 24650
Closed treatment of radial head or neck fracture; with manipulation	CPT 24655
CPT, Current Procedural Terminology (American Medical Association, Chicago, IL, USA); ICD-9, International Classification of Diseases, Ninth Edition. *These codes were used in the query of both ORIF for Olecranon fractures and ORIF of radial head and neck fractures.	

**Table 2:** Annual RHNF incidence and management among the Humana population between 2007 and 2014.

Year	RHNF	RHA	% Use of RHA	ORIF	% Use of ORIF	Non-op Tx	% Use of Non-op Tx
**2007**	1,773	77	4.34%	545	30.74%	550	31.02%
**2008**	2,020	88	4.36%	584	28.91%	574	28.42%
**2009**	2,122	102	4.81%	676	31.86%	673	31.72%
**2010**	2,319	93	4.01%	671	28.93%	684	29.50%
**2011**	2,462	129	5.24%	704	28.59%	737	29.94%
**2012**	2,631	141	5.36%	723	27.48%	771	29.30%
**2013**	3,156	182	5.77%	785	24.87%	885	28.04%
**2014**	3,917	216	5.51%	924	23.59%	1,013	25.86%
**Totals**	**20,400**	**1,028**		**5,612**		**5,887**	
***Trend*** ***p-value***	***<0.001***	***<0.001***	***0.011***	***<0.001***	***0.004***	***<0.001***	***0.046***

**Table 3:** Annual OF incidence and management among the Humana population between 2007 and 2014.

Year	**Incidence of OF**	ORIF	% Use of ORIF	**Non-op Tx**	% Use of Non-op Tx
**2007**	948	726	76.58%	98	10.34%
**2008**	1,030	787	76.41%	102	9.90%
**2009**	1,098	910	82.88%	112	10.20%
**2010**	1,261	968	76.76%	144	11.42%
**2011**	1,312	1,040	79.27%	148	11.28%
**2012**	1,287	1,070	83.14%	169	13.13%
**2013**	1,649	1,227	74.41%	218	13.22%
**2014**	2,024	1,418	70.06%	234	11.56%
**Totals**	**10,609**	**8,146**		**1,225**	
*Annual trend p-value*	*0.001*	*<0.001*	*0.392*	*<0.001*	*0.022*

**Table 4:** Summary of study findings that reshaped the management of radial head fractures.

Method of treatment discussed	Authors	Population	Summary of key findings
Radial head resection	Hall *et al.* [24]	Case series: 42 patients evaluated for elbow or forearm problems after radial head resection.	7 patients (17%) were diagnosed with posterolateral rotational instability of the elbow.
Non-operative	Akesson *et al.* [20]	49 patients treated non-operatively for Mason type-IIa radial head fractures. 6 patients had delayed radial head excision for poor outcome.	40 of the 49 patients (82%) had no subjective complaints after non-operative treatment. Injured elbows had significantly lower ROM and higher degenerative changes compared to non-injured elbows but this was thought to be clinically insignificant by the authors.
	Herbertsson *et al.* [25]	Retrospective study: 100 patients treated non-operatively, with radial head resection, or with ORIF for Mason type-II or III radial head and neck fractures. Mean follow-up was 19 years.	77 of the 100 patients had no symptoms at follow-up. Mean ROM deficits were minor (2 degree flexion, 3 degree extension, and 3 degree supination deficits). 19 patients had primary radial head excision while 9 patients had a secondary procedure performed due to residual pain.
ORIF	Ring *et al.* [19]	Retrospective study: 56 patients treated with ORIF for intra-articular radial head fracture.	14 out of 15 patients with Mason Type-3 comminuted radial head fractures with at least 3 articular fragments had unsatisfactory results (based on failure of fixation or nonunion requiring radial head excision, a fair or poor result with the system of Broberg and Morrey, or recovery of < 100 degrees of forearm rotation).
RHA	Yan *et al.* [27]	RCT: 39 patients with Mason type-3 radial head fractures and associated terrible triad injuries of the elbow	RHA group had a significant shorter surgery duration, fewer post-surgical complications, and better clinical outcomes compared to the ORIF group.
	Chen *et al.* [26]	RCT: 45 patients with unstable, multi-fragmented fractures of the radial head treated with either ORIF or RHA	Better outcome scores and lower complication rates among the RHA group compared to the ORIF group.

**Table 5:** Summary of study findings that reshaped the management of olecranon fractures in elderly patients.

Authors	Population	Summary of key findings
Veras Del Monte *et al.* [14]	Retrospective study: 12 elderly patients (mean age 81.8 years) treated non-operatively for displaced olecranon fractures.	92% of patients had excellent satisfaction and 67% of patients reported no pain.
Duckworth *et al.* [12]	Trauma database study: 43 older patients (mean age 76 years) treated non-operatively for olecranon fractures.	Short-term follow-up (mean 4 months): 72% of patients had excellent or good outcomes. Long-term follow-up (mean 6 years): Overall patient satisfaction rating of 91%.
Gallucci *et al.* [13]	Retrospective study: 28 elderly patients (mean age 82 years) treated non-operatively for displaced olecranon fractures.	Median VAS pain score was 1.1 out of 10 and the mean satisfaction score was 9 out of 10. Non-union occurred in 85% of patients but the authors thought this to be irrelevant to final outcomes.

## LIST OF ABBREVIATIONS

OF: = Olecranon fractures
ORIF: = Open reduction and internal fixation
RHA: = Radial head arthroplasty
RHNF: = Radial head and neck fractures
